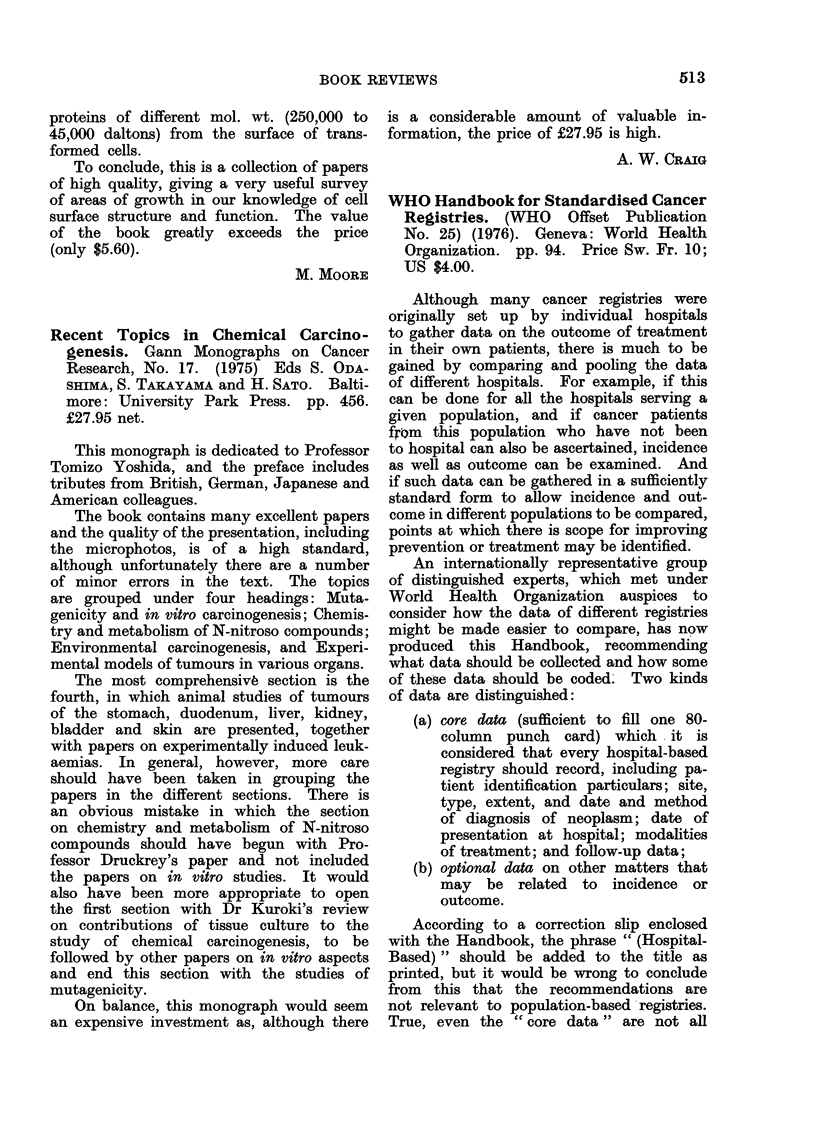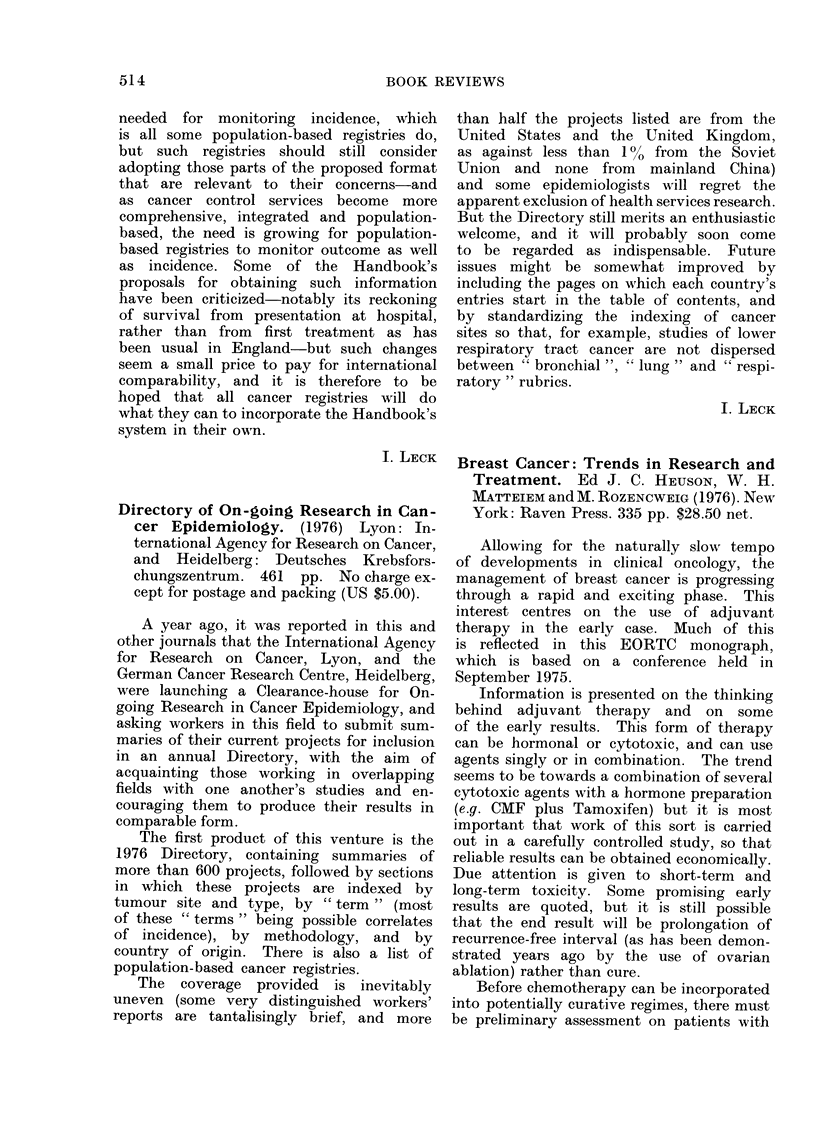# WHO Handbook for Standardised Cancer Registries

**Published:** 1977-04

**Authors:** I. Leck


**WHO Handbook for Standardised Cancer Registries.** (WHO Offset Publication No. 25) (1976). Geneva: World Health Organization. pp. 94. Price Sw. Fr. 10; US $4.00.

Although many cancer registries were originally set up by individual hospitals to gather data on the outcome of treatment in their own patients, there is much to be gained by comparing and pooling the data of different hospitals. For example, if this can be done for all the hospitals serving a given population, and if cancer patients from this population who have not been to hospital can also be ascertained, incidence as well as outcome can be examined. And if such data can be gathered in a sufficiently standard form to allow incidence and outcome in different populations to be compared, points at which there is scope for improving prevention or treatment may be identified.

An internationally representative group of distinguished experts, which met under World Health Organization auspices to consider how the data of different registries might be made easier to compare, has now produced this Handbook, recommending what data should be collected and how some of these data should be coded. Two kinds of data are distinguished:

(a) *core data* (sufficient to fill one 80-column punch card) which it is considered that every hospital-based registry should record, including patient identification particulars; site, type, extent, and date and method of diagnosis of neoplasm; date of presentation at hospital; modalities of treatment; and follow-up data;

(b) *optional data* on other matters that may be related to incidence or outcome.

According to a correction slip enclosed with the Handbook, the phrase " (Hospital-Based) " should be added to the title as printed, but it would be wrong to conclude from this that the recommendations are not relevant to population-based registries. True, even the " core data " are not all needed for monitoring incidence, which is all some population-based registries do, but such registries should still consider adopting those parts of the proposed format that are relevant to their concerns—and as cancer control services become more comprehensive, integrated and population-based, the need is growing for population-based registries to monitor outcome as well as incidence. Some of the Handbook's proposals for obtaining such information have been criticized—notably its reckoning of survival from presentation at hospital, rather than from first treatment as has been usual in England—but such changes seem a small price to pay for international comparability, and it is therefore to be hoped that all cancer registries will do what they can to incorporate the Handbook's system in their own.

I. LECK